# Supplementary material for: The genome sequence of the Powdered Quaker, Orthosia gracilis (Schiffermüller, 1775)
Source: Wellcome Open Res. 2023 Jul 7;8:290. [Version 1] doi: 10.12688/wellcomeopenres.19644.1 (PMC10448147; doi:10.12688/wellcomeopenres.19644.1)
Supplement: Peer review history

# Peer review: Reviewer response for version 1

**DOI:** 10.21956/wellcomeopenres.21761.r65317

**Published:** 2023-08-23

**Authors:** Joel Vizueta

**Affiliations:** 1 University of Copenhagen, Copenhagen, Capital Region of Denmark, Denmark

The authors describe in this genome report the chromosome level assembly of the powdered quaker *Orthosia gracilis*. The sequenced genome is very high quality based on the reported metrics, and the methods are robust. This genomic resource will be interesting in comparative genomics analyses within Lepidoptera, and the presented data is already publicly available. 

 Based on current genome reports published in Wellcome Open Research from the Darwin Tree of Life, this article follows the same structure and contents, therefore I would recommend this manuscript to be accepted as it is.

Are sufficient details of methods and materials provided to allow replication by others?

Yes

Is the rationale for creating the dataset(s) clearly described?

Yes

Are the datasets clearly presented in a useable and accessible format?

Yes

Are the protocols appropriate and is the work technically sound?

Yes

Reviewer Expertise:

Ecology and evolution; Comparative genomics

I confirm that I have read this submission and believe that I have an appropriate level of expertise to confirm that it is of an acceptable scientific standard.

# Peer review: Reviewer response for version 1

**DOI:** 10.21956/wellcomeopenres.21761.r64227

**Published:** 2023-08-22

**Authors:** Niklas Wahlberg

**Affiliations:** 1 Lund university, Lund, Sweden

This is yet one more Lepidoptera genome put together by the Darwin Tree of Life project. As there are already several hundred such genomes and genome reports, I expect that the major wrinkles in protocols and how to report them have been ironed out. And so it would seem with this genome report. The genome of *Orthosia gracilis* falls within the expected size of species in the so-called "pest clade" of Noctuidae. With 50 Noctuidae genomes already published, and another 90 or so publicly available, this family is poised to become a standard for studying genome evolution in insects. Not much else to say, other than the *Orthosia gracilis* genome looks ready to be used in further analyses!

Are sufficient details of methods and materials provided to allow replication by others?

Yes

Is the rationale for creating the dataset(s) clearly described?

Yes

Are the datasets clearly presented in a useable and accessible format?

Yes

Are the protocols appropriate and is the work technically sound?

Yes

Reviewer Expertise:

Phylogenomics of Lepidoptera, systematics, molecular phylogenetics.

I confirm that I have read this submission and believe that I have an appropriate level of expertise to confirm that it is of an acceptable scientific standard.
